# Cross-cultural adaptation and validation of a Bengali version of the modified fibromyalgia impact questionnaire

**DOI:** 10.1186/1471-2474-13-157

**Published:** 2012-08-27

**Authors:** Mohammed A Muquith, Md Nazrul Islam, Syed A Haq, Peter M ten Klooster, Johannes J Rasker, Muhammad B Yunus

**Affiliations:** 1 Rheumatology Wing, Department of Medicine, Bangabandhu Sheikh Mujib, Medical University, Shahbagh, Dhaka, Bangladesh; 2 Department of Psychology, Health & Technology, University of Twente, Enschede, The Netherlands; 3 Section of Rheumatology, The University of Illinois College of Medicine, Peoria, IL, USA

**Keywords:** FIQ, Validation, Fibromyalgia, Reliability, Bengali, Questionnaire

## Abstract

**Background:**

Currently, no validated instruments are available to measure the health status of Bangladeshi patients with fibromyalgia (FM). The aims of this study were to cross-culturally adapt the modified Fibromyalgia Impact Questionnaire (FIQ) into Bengali (B-FIQ) and to test its validity and reliability in Bangladeshi patients with FM.

**Methods:**

The FIQ was translated following cross-cultural adaptation guidelines and pretested in 30 female patients with FM. Next, the adapted B-FIQ was physician-administered to 102 consecutive female FM patients together with the Health Assessment Questionnaire (HAQ), selected subscales of the SF-36, and visual analog scales for current clinical symptoms. A tender point count (TPC) was performed by an experienced rheumatologist. Forty randomly selected patients completed the B-FIQ again after 7 days. Two control groups of 50 healthy people and 50 rheumatoid arthritis (RA) patients also completed the B-FIQ.

**Results:**

For the final B-FIQ, five physical function sub-items were replaced with culturally appropriate equivalents. Internal consistency was adequate for both the 11-item physical function subscale (α = 0.73) and the total scale (α = 0.83). With exception of the physical function subscale, expected correlations were generally observed between the B-FIQ items and selected subscales of the SF-36, HAQ, clinical symptoms, and TPC. The B-FIQ was able to discriminate between FM patients and healthy controls and between FM patients and RA patients. Test-retest reliability was adequate for the physical function subscale (r = 0.86) and individual items (r = 0.73-0.86), except anxiety (r = 0.27) and morning tiredness (r = 0.64).

**Conclusion:**

This study supports the reliability and validity of the B-FIQ as a measure of functional disability and health status in Bangladeshi women with FM.

## Background

Fibromyalgia is a common disorder characterized by widespread musculoskeletal pain, stiffness, paraesthesia, non-restorative sleep, and fatigue along with multiple tender points, which are widely and symmetrically distributed [1]. FM is an important cause of disability and work absenteeism and has major personal, social, and economic consequences [2,3]. It affects at least 2% of the general population in the United States [4] and a similar prevalence exist worldwide [5]. It is also common in Bangladesh with a point prevalence of 4.4% [6]. Approximately 6-10% of all individuals in a physician’s waiting room have FM [7], so most physicians can expect to encounter at least one patient with FM daily.

There are several instruments available to measure the health status and physical function of patients with rheumatic diseases in general, including the Health Assessment Questionnaire (HAQ) [8] and the Arthritis Impact Measurement Scales (AIMS) [9]. Because these questionnaires do not fully reflect the multidimensionality of FM symptoms, the Fibromyalgia Impact Questionnaire (FIQ) was developed to specifically capture the total spectrum of problems related to FM [10,11]. Since its introduction in 1991 [10], the FIQ has become a widely used and popular instrument in FM research. The scale has been translated and validated in several languages, including Hebrew, Swedish, Korean, German, French, Spanish, Italian, Turkish, and Dutch [12-21]. Overall, it has shown credible construct validity, adequate reliability, and excellent responsiveness to change and has been recommended as a primary endpoint in FM clinical trials [11,22].

To date, however, the FIQ has not been translated into Bengali. With nearly 300 million speakers, Bengali is the sixth language in the world, second in India and the national language in Bangladesh [23]. Therefore, the aim of the present study was to cross-culturally adapt the FIQ into Bengali and to evaluate its reliability and construct validity in Bangladeshi patients with fibromyalgia.

## Methods

The original US modified version of the FIQ consists of 10 items [11]. The first item contains 11 sub-items measuring the ability to perform activities of daily living in the past week on a 4-point Likert-type scale from 0 (always) to 3 (never) and is referred to as the physical function subscale. Item 2 and 3 ask for the number of days felt good and the number of days (0–7) unable to work during the past week. Items 4–10 are horizontal linear scales marked in 10 increments on which the patient rates work difficulty, pain, fatigue, morning tiredness, stiffness, anxiety, and depression in the past week. Scores on each of the 10 items are standardized so as to range from 0 to 10, with higher scores indicating greater impairment. The total score can range from 0 to 100.

### Cross-cultural translation of the FIQ

Cross-cultural adaptation was carried out in five stages according to the recommendations by Beaton et al. [24].

#### Stage I - III. Forward and backward translation

The forward translation was performed by two bilingual translators with different profiles whose mother tongue was Bengali. One of the translators with a medical background was aware of the concepts being examined in the questionnaire. The other, a university business administration student, was not aware or informed of the concepts being quantified. After this, both translators and three of the authors sat down to produce a synthesis version from the original questionnaire as well as both translations. The synthesis version was back translated into English by two independent translators proficient in English and without any medical background. One of them was a University lecturer in English and the other was a professional translator. Both were not aware or informed of the concepts explored.

#### Stage IV. Expert committee

An expert committee was formed which included health professionals, methodologists, and the translators involved in the process. The committee reviewed all translations and the original questionnaire to reach consensus on any discrepancy and to produce a preliminary version for field testing. Several critical decisions were made to achieve semantic, idiomatic, experiential, and conceptual equivalence between the original and back-translated versions. For some physical function sub-items that refer to activities not commonly practiced by Bengali women, such as using a washer and dryer and vacuuming a rug, the committee added culturally appropriate equivalents (Table 1). This resulted in a preliminary Bengali version of the FIQ that included 20 original and 11 added or modified sub-items for the physical function subscale.

**Table 1:** Original and added equivalent items of the physical function subscale used in pretesting the preliminary version in FM patients (n = 30)

**#**	**Item**	**Impairment**	No impairment**
1	Do shopping?	22 (73,3%)	8 (26.7%)
2	Do laundry with washer and dryer?	1 (3.3%)	29 (96.7%)
2.1*	Wash clothes?	29 (96.7%)	1 (3.3%)
2.2*	Operate sewing machine by hand?	4 (13.3%)	26 (86.7%)
3	Prepare meals?	29 (96.7%)	1 (3.3%)
4	Wash dishes/ cooking utensils by hand?	27 (90%)	3 (30%)
5	Vacuum a rug?	1 (3.3%)	29 (96.7%)
5.1*	Sweep floor with a broom?	28 (93.3%)	2 (6.7%)
5.2*	Pray in usual way?	28 (93.3%)	2 (6.7%)
6	Make beds?	30 (100%)	0 (0%)
6.1*	Sew with needle and thread?	7 (23.3%)	23 (76.7%)
7	Walk several blocks?	0 (0%)	30 (100%)
7.1*	Walk more than 1 km?	21 (70%)	9 (30%)
8	Visit friends or relatives?	24 (80%)	6 (20%)
9	Do yard work?	0 (0%)	30 (100%)
9.1*	Clean (including sweeping) the yard?	13 (43.3%)	17 (56.7%)
9.2*	Work in the field?	0 (0%)	30 (100%)
9.3*	Work in garden in front of your house?	4 (13.3%)	26 (86.7%)
10	Drive a car?	0 (0%)	30 (100%)
10.1*	Dress vegetable with the help of a ‘boti’?	30 (100%)	0 (0%)
11	Climb stairs?	28 (93.3%)	2 (6.7%)
11.1*	Climb up the ‘ghat’?	7 (23.3%)	23 (76.7%)

*Added equivalent items; **Occasionally or never. Note: a Boti is a special cutting instrument that is bent upwards. A Ghat is the modified bank of a river or pond used for washing, ablution etc.

#### Stage V: Pretest

The preliminary version of the Bengali FIQ (B-FIQ) was field tested in 30 female patients fulfilling the ACR criteria for FM [25]. Since only two patients were able to independently complete the questionnaire, the questionnaire was subsequently administered in a face-to-face interview. Consecutive FM out-patients who consented to participate were enrolled from the rheumatology wing of the Bangabandhu Sheikh Mujib Medical University (BSMMU). To test for face and content validity, patients were probed about what they thought was meant by each item and the chosen response. Additionally, patients were asked if they were accustomed to performing the activities.

### Psychometric evaluation of the B-FIQ

#### Participants

A new group of 102 adult female FM patients fulfilling the 1990 ACR criteria for FM [25] were enrolled consecutively from the BSMMU and a satellite clinic of the Community Oriented Program for Control of Rheumatic Disorders (COPCORD) at Sonargaon, Narayangonj, Bangladesh. Patients with a history of autoimmune disease or inflammatory arthritis were excluded. A random sample of 40 patients was requested to visit the clinic again 7 days after the first visit. During that week no intervention was given. Additionally, two control groups matched for age and sex of 50 patients with rheumatoid arthritis (RA) and 50 healthy persons each were enrolled. The healthy controls were a convenience sample of people without musculoskeletal complaints. The study protocol was approved by the ethics committee of the BSMMU, Dhaka, Bangladesh. All participants provided verbal informed consent and completed the pre-final B-FIQ and socio-demographic items in a face-to-face interview. The time it took to administer the B-FIQ was recorded in both the FM patients and healthy controls.

#### Additional measures

FM patients were additionally administered a validated Bengali version of the HAQ [26] and relevant mental health subscales of the SF-36 (general health, vitality, role-emotional, and mental health) [27]. To assess current disease symptoms, pain, morning stiffness, fatigue, sleep disturbance, headache problem, and depression were recorded on 100 mm visual analog scales (VASs) with 100 denoting the worst possible condition. Also, a tender point count (TPC) was performed by a rheumatologist. Tenderness was assessed by applying about 4 kg finger pressure at all tender points until the fingernail bed blanched [25].

#### Statistical analysis

To assess the content validity of the physical function sub-items, a cutoff criterion of ≥25% impairment response (‘occasionally’ or ‘never’) was set to indicate a valid item [10,13]. Additionally, missing values were examined. Internal consistency of the physical function subscale and the total B-FIQ was assessed by Cronbach’s α, where a value ≥0.70 was considered adequate for group comparisons and a value between 0.90–0.95 for individual comparisons [28]. Construct validity of the B-FIQ was assessed by examining Spearman correlations between the B-FIQ scores and clinical symptom severity VASs, scores on the HAQ and relevant mental scales of the SF-36, and the TPC [12-14,21]. Known groups validity was assessed by comparing B-FIQ scores of the FM patients with those of the healthy controls and RA patients [14] using Mann–Whitney U tests. Finally, test-retest reliability was assessed by computing Spearman correlations between the B-FIQ scores of the 40 FM patients that completed the B-FIQ again after 7 days. As with internal consistency, test-retest reliability coefficients ≥0.70 and between 0.90–0.95 were considered adequate for group level and individual measurements over time, respectively [28]. All statistical analyses were performed using SPSS for Windows version 11.5.

## Results

### Pretesting of the FIQ

Mean age of the 30 patients included in the pretest was 32.1 ± 11.3 years and 53.3% and 33.3% were housewife or student, respectively. 23.3% had no education, whereas 20% and 43.3% had followed primary or secondary education, respectively. From the 22 physical function sub-items (Table 1), four original items (items 2, 5, 7, and 9) were not understood by most of the patients. The remaining original and all added equivalent sub-items were understood by the patients, although many patients were not accustomed to doing 6 of the activities mentioned (items 2.2, 6.1, 9.2, 9.3, 10, and 11.1). The remaining 12 sub-items were kept for the pre-final questionnaire with some clarification added to some items based on the comments made by the patients (items 1, 2.1, 6, and 8). As patients mentioned practicing different religions, item 5.2 (“Pray in usual way”) was changed into “Do worship / religious prayer in usual way”.

### Psychometric evaluation of the B-FIQ

Descriptive characteristics of the included participants are listed in Table 2. The three samples were comparable regarding age, occupation, religion, and marital status. However, there were significant differences in educational level. On average, 9.6 ± 1.6 minutes were needed to administer the B-FIQ to the FM patients, whereas it took a mean time of 7.4 ± 1.3 minutes to administer the B-FIQ to the healthy controls (P difference <0.001).

**Table 2:** Descriptive characteristics of the FM patients, healthy controls, and RA patients

**Variable**	**FM patients**	**Healthy controls**	**RA patients**	P*
	**(n = 102)**	**(n = 50)**	**(n = 50)**	
Age (years)	32.1 ± 11.8	33.6 ± 12	35.9 ± 11.1	0.171
Disease duration (months)	31.5 ± 3.2	-	-	-
Occupation, n (%)				0.052
Housewife	67 (65.7)	28 (56.0)	39 (78.0)	
Student	16 (15.7)	5 (10.0)	5 (10.0)	
Other	19 (18.6)	17 (34.0)	6 (12)	
Religion, n (%)				0.078
Islam	100 (98.0)	47 (94.0)	45 (90.0)	
Hinduism/Buddhism	2 (2.0)	3 (6.0)	5 (10.0)	
Marital status, n (%)				0.101
Married	71 (69.6)	32 (64.0)	33 (66.0)	
Unmarried	22 (21.6)	13 (26.0)	6 (12.0)	
Widow/divorced	9 (8.8)	5 (10.0)	11 (22.0)	
Educational level, n (%)				0.012
No education	18 (17.6)	18 (36.0)	14 (28.0)	
Primary	28 (27.7)	9 (18.0)	10 (20.0)	
Secondary	48 (47.1)	12 (24.0)	19 (38.0)	
Graduate and above	8 (7.8)	11 (22.0)	7 (14.0)	

Values are mean ± SD or n (%). *One-way ANOVA, chi-square test or Fisher’s exact test where appropriate.

Using the cutoff of ≥25% impairment, all 12 physical function sub-items met the criterion. Also, missing data within the physical function subscale of the B-FIQ were limited to 2.9% of the patients who did not “Dress vegetable with the help of a boti” (item 10) and 53.9% of the patients who did not “Clean (including sweeping) the yard” (item 9), suggesting adequate content validity of the majority of physical activities.

The internal consistency of the initial 12 sub-items constituting the physical function subscale was inadequate, with Cronbach’s α = 0.67. Removing the item “Do worship / religious prayer in usual way,” which was tested as a second possible equivalent to “vacuum a rug” raised Cronbach’s α to an acceptable level of 0.73. Consequently, this item was removed from the scale for the further analyses and the final B-FIQ (Additional file 1), also making the B-FIQ more comparable to the original US FIQ. Cronbach’s α for all 10 individual items of the total B-FIQ was 0.83, also indicating acceptable internal consistency for group level analyses.

As expected, B-FIQ scores were significantly correlated in the expected direction with most current FM symptoms recorded on the VASs (Table 3). Correlations were generally strong for similar aspects of health and moderate for different aspects of health. Exceptions were physical function, which correlated only with tiredness on the VAS and the TPC, and workdays missed, which correlated only with the TPC. Correlations with overall disease severity were moderate and correlations with the TPC weak to moderate. Additionally, the B-FIQ items were significantly correlated with all selected subscales from the HAQ and the SF-36, except for a lack of correlation between workdays missed and general health (Table 4). Correlations were generally strongest for similar aspects of health, such as morning tiredness vs. vitality and anxiety and depression vs. mental health. However, physical function was only weakly correlated with the HAQ disability index.

**Table 3:** Spearman correlations with other measures in FM patients (n = 102)

**B-FIQ questions**	**Current FM symptoms (VAS)**							**TPC**
	**Pain**	**Tiredness**	**Stiffness**	**Depression**	**Sleep problems**	**Headache**	**Disease severity**	
Physical function	0.026	0.216*	0.176	0.187	0.142	0.105	0.136	0.245*
Days felt good	0.301**	0.317**	0.241*	0.330**	0.335**	0.281**	0.293**	0.217*
Workdays missed	0.146	0.124	0.114	0.173	0.112	0.060	0.097	0.246*
Ability to do job	0.559**	0.418**	0.433**	0.521**	0.454**	0.364**	0.422**	0.382**
Pain	0.750**	0.333**	0.289**	0.417**	0.296**	0.220*	0.437**	0.247*
Fatigue	0.371**	0.566**	0.479**	0.286**	0.370**	0.465**	0.445**	0.274**
Morning tiredness	0.405**	0.923**	0.359**	0.473**	0.502**	0.408**	0.545**	0.327**
Stiffness	0.345**	0.266**	0.780**	0.300**	0.371**	0.365**	0.358**	0.153
Anxiety	0.465**	0.419**	0.340**	0.579**	0.372**	0.272**	0.415**	0.387**
Depression	0.324**	0.559**	0.260**	0.790**	0.318**	0.271**	0.544**	0.288**

*VAS* = visual analog scale; *TPC* = tender point count. * P < 0.05; ** P < 0.01.

**Table 4:** Spearman correlations with selected measures in FM patients (n = 102)

**B-FIQ questions**	**Measure**	**Subscale**	**r**	**P**
Physical function	HAQ	Disability index	0.236	0.021
	SF-36	Vitality	- 0.239	0.016
Days felt good	SF-36	General health	- 0.245	0.013
		Mental health	- 0.303	0.002
Workdays missed	HAQ	Disability index	0.281	0.006
	SF-36	Vitality	- 0.224	0.023
		General health	- 0.123	0.218
Ability to do job	SF-36	Vitality	- 0.405	<0.001
	HAQ	Disability index	0.186	0.070
Pain	HAQ	Disability index	0.265	0.009
Fatigue	SF-36	Vitality	- 0.393	<0.001
Morning tiredness	SF-36	Vitality	- 0.598	<0.001
Stiffness	HAQ	Disability index	0.284	0.005
Anxiety	SF-36	Role-emotional	- 0.360	<0.001
		Mental health	- 0.439	<0.001
Depression	SF-36	Role-emotional	- 0.280	0.004
		Mental health	- 0.494	<0.001

*HAQ* = Health Assessment Questionnaire; *SF-36* = Short Form 36 health survey.

FM patients scored significantly worse than healthy controls on all items of the B-FIQ (Table 5). The mean total B-FIQ score was more than 6 times higher in FM patients than in healthy controls. The B-FIQ was also able to discriminate between FM and RA patients, as demonstrated by significantly higher scores for FM patients on all items except number of workdays missed. Also, the mean total B-FIQ score was substantively higher in FM patients compared to RA patients.

**Table 5:** Discriminant validity of the B-FIQ in FM patients, healthy controls, and RA patients

**FIQ questions**	**Group**		P*	**Group**		P*
	**FM patients**	**Healthy controls**		**FM patients**	**RA patients**	
	**(n = 102)**	**(n = 50)**		**(n = 102)**	**(n = 50)**	
Physical function	4.96 ± 2.47	0.92 ± 0.90	<0.001	4.96 ± 2.47	3.30 ± 0.43	<0.001
Days felt good	5.03 ± 2.09	0.20 ± 0.63	<0.001	5.03 ± 2.09	2.54 ± 0.41	<0.001
Workdays missed	1.72 ± 0.24	0.0 ± 0.0	<0.001	1.72 ± 0.24	1.32 ± 0.35	0.150
Ability to do job	7.26 ± 2.49	0.28 ± 0.11	<0.001	7.26 ± 2.49	3.98 ± 0.47	<0.001
Pain	7.55 ± 2.27	0.95 ± 0.16	<0.001	7.55 ± 2.27	3.51 ± 0.42	<0.001
Morning tiredness	8.02 ± 2.69	1.70 ± 0.26	<0.001	8.02 ± 2.69	4.64 ± 0.48	<0.001
Fatigue	6.89 ± 2.98	0.43 ± 0.17	<0.001	6.89 ± 2.98	3.54 ± 0.48	<0.001
Stiffness	5.22 ± 3.30	0.30 ± 0.13	<0.001	5.22 ± 3.30	3.53 ± 0.46	0.005
Anxiety	7.72 ± 2.75	2.39 ± 0.33	<0.001	7.72 ± 2.75	5.07 ± 0.50	<0.001
Depression	7.21 ± 2.80	2.46 ± 0.29	<0.001	7.21 ± 2.80	4.40 ± 0.45	<0.001
Total	64.75 ± 16.47	9.67 ± 6.69	<0.001	64.75 ± 16.47	37.56 ± 3.66	<0.001

* Mann–Whitney *U* test.

Mean scores of the 40 patients that completed the B-FIQ twice and test-retest correlations are presented in Table 6. Test-retest correlations were sufficiently high for group level comparisons for all items, except for anxiety and morning tiredness.

**Table 6:** One-week test-retest reliability of the B-FIQ in FM patients (n = 40)

**FIQ item**	**B-FIQ score**		**Spearman’s r**
	**Time 1**	**Time 2**	
Physical function	4.8 ± 2.5	5.2 ± 2.1	0.860
Days felt good	5.1 ± 2.1	4.8 ± 2.2	0.773
Workdays missed	2.0 ± 0.4	1.7 ± 0.4	0.862
Ability to do job	6.7 ± 2.3	7.2 ± 2.1	0.809
Pain	7.0 ± 2.3	6.9 ± 2.4	0.773
Morning tiredness	7.8 ± 2.7	7.3 ± 2.6	0.643
Fatigue	6.8 ± 2.9	6.5 ± 2.9	0.887
Stiffness	4.6 ± 3.5	5.1 ± 3.4	0.793
Anxiety	7.4 ± 2.7	9.6 ± 2.4	0.265
Depression	6.7 ± 3.0	7.1 ± 2.9	0.733
Total	61.9 ± 15.7	62.9 ± 16.0	0.899

## Discussion

Tools for evaluating health status have been developed mainly for use in English-speaking countries [29,30]. In order to assess the health status of different cultural groups and to compare the results of trials in different countries, the need for non-English language measures has increased [31]. To date, no validated disease-specific measure was available to assess the full spectrum of FM problems in Bengali patients. In this study, we developed and evaluated a cross-culturally adapted version of the FIQ for use in Bengali-speaking patients with FM. Overall, the findings suggest that the B-FIQ is a sufficiently reliable and valid measure of health status in Bangladeshi women with FM. However, some limitations were apparent that need further study, including the low correlation between the physical function scale and similar or conceptually related scales and the low test-retest reliability of the anxiety and morning tiredness items.

Cross-cultural adaption of a questionnaire is more challenging than merely translating its items into another language. To be used across cultures, the items must not only be translated well linguistically, but also have to be adapted to the specific culture to maintain content validity at a conceptual level [24]. This may involve changing or replacing items that are not experienced in the target culture [24,31]. To adapt the FIQ for use in Bengali patients, five sub-items of the final B-FIQ physical function scale (i.e., do laundry with a washer and dryer, vacuum a rug, walk several blocks, do yard work, and drive a car) were replaced with culturally appropriate equivalent activities since these activities are not commonly performed or understood by Bengali women. Similar modifications have been made to these items in previous cultural adaptations of the FIQ [12,14,21]. Additionally, several other physical function items were slightly modified or clarified. The finding that especially the sub-items of the physical function subscale needed adaptations corresponds with findings from several translations of the widely used SF-36 health status measure, which also showed that the most difficult items to translate were physical functioning items that refer to activities not common outside the US [31]. In 2009, Bennett et al. [32] developed a revised version (the FIQR) in response to several deficiencies, including the fact that the functional questions were originally intended for women living in reasonably affluent countries. It is likely that the new physical function items of the FIQR are more appropriate to Bengali women as well and could provide better cross-cultural compatibility. Therefore, it would be worthwhile for future studies to validate the FIQR in Bangladeshi patients with FM.

During the pre-testing phase, it became clear that most Bengali FM patients were not able to complete the questionnaire by themselves. Although the FIQ was developed as a self-administered questionnaire, we decided to administer the questionnaire in a face-to-face interview context. This inability to self-complete the questionnaire was most likely due to patients’ lack of previous experience with research and participation in such studies. Most patients were not familiar with completing questionnaires and scoring VASs. Also, the low literacy rate among Bangladeshi women may have contributed.

Additionally, there was a notable difference in the time it took to administer the B-FIQ to the FM patients and the healthy controls in the psychometric evaluation study. The longer administration time in the FM patients may be the result of more cognitive dysfunction in this group, which is increasingly recognized as a key symptom of FM [33,34].

The internal consistencies of the physical function scale and the total B-FIQ were adequate for group level comparisons and suggest that scores on the sub-items of the physical function scale and the scores on all 10 items of the B-FIQ can be summed to create single total scores. With a Cronbach’s α of 0.83, the internal consistency of the total scale was comparable with previous translations of the FIQ, where α’s have ranged between 0.72 and 0.93 [13,15,17-19,21]. Internal consistency of the physical functioning scale (α = 0.73) was somewhat lower than the values of 0.86 and 0.91 found in previous studies [17,20]. Since one item was removed from the pre-final B-FIQ due to its low item-total correlation, the final B-FIQ consists of 11 items similar which are similar in content to the original US version.

The significant correlations between most FIQ items and other outcome measures suggest that the FIQ has adequate construct validity. Most notable exceptions were physical function, which only correlated with tiredness and the TPC, and workdays missed, which only correlated with the TPC. Additional analyses using selected scales from the HAQ and SF-36 as convergent measures showed that both were significantly, but only weakly, related to the HAQ disability index and the SF-36 vitality scale.

The finding that workdays missed does not correlate well with many other outcomes was also apparent in previous translation studies [14,17,20,21]. This difference with the original US questionnaire may be the result of cultural differences in employment and working conditions between countries. In their evaluation study of the Dutch FIQ, for instance, Zijlstra et al. [20] argued that the low correlations were probably due to the small number of women who had a job. In the Bengali socio-cultural situation, in particular, women often cannot skip work, especially housework, even if they feel very sick. The non-significant or weak correlations between the physical functioning scale and most other measures does not correspond with most previous studies. Although in the Korean version of the FIQ this item also correlated poorly with current FM symptoms [21], most studies did find moderate to high correlations with concurrent measures such as the HAQ and the SF-36 physical functioning scale [13-15,17,20,21].

Except for stiffness, all B-FIQ items were significantly but only weakly or moderately correlated with the TPC. This is in accordance with previous studies that also showed weak [10,16,17,21] or moderate [13,15,18,19] correlations between FIQ items and TPCs. It is also in line with findings by Jacobs et al. [35], who found a weak correlation between TPCs and self-reported pain. They concluded that TPCs and self-reported pain represent different aspects of pain in FM. Callahan and Pincus even suggested that this is a specific feature of FM [36].

The B-FIQ was highly capable of discriminating between FM patients and healthy controls and between FM patients and RA patients. Score differences between FM patients and RA patients were significant for all items except workdays missed. This is consistent with the findings by Hedin et al. in Sweden [14]. FM patients also scored worse on the number of days felt good, suggesting that the FIQ may be a more appropriate instrument for evaluating FM patients than the HAQ or AIMS.

Finally, test-retest reliability was adequate for all B-FIQ items except morning tiredness and anxiety. Low test-retest coefficients have been previously reported for morning tiredness [17,20,21], but not for anxiety. Other studies, however, did report low reliably for varying items of the FIQ [12,14,16]. Perrot et al. [16] have suggested that this may be due to the variability of the multiple aspects of the FM syndrome.

## Conclusions

The results of this study suggest that the B-FIQ is a reliable and valid measure of health status for female FM patients in Bangladesh. Future studies should further examine the issues related to the construct validity of the physical function subscale and the reliability of the anxiety item. Additionally, the responsiveness of the B-FIQ should be examined in longitudinal clinical trials.

## Competing interests

The authors declare that they have no competing interests.

## Authors’ contributions

MAM, MNI, SAH, and MBY conceived and designed the study. MAM collected the data. MAM, MNI, JJR and PTK performed the statistical analyses and interpretation of the data. MAM, MNI and PTK drafted the manuscript. MNI, SAQ, JJR and MBY supervised the study. All authors critically reviewed, contributed to and approved the final manuscript.

## Pre-publication history

The pre-publication history for this paper can be accessed here:

http://www.biomedcentral.com/1471-2474/13/157/prepub

## Supplementary Material

Additional file 1 Final version of the B-FIQ (Bengali and English items). Click here for file

## References

[B1] Wolfe F Rasker JJ Firestein GS, Budd RC, Harris ED Jr, McInnes IB, Ruddy S, Sergent JS Fibromyalgia Kelley's Textbook of Rheumatology 2008 8 Philadelphia, PA: Saunders/Elsevier 555 570

[B2] Boonen A van den Heuvel R van Tubergen A Goossens M Severens JL van der Heijde D van der Linden S Large differences in cost of illness and wellbeing between patients with fibromyalgia, chronic low back pain, or ankylosing spondylitis Ann Rheum Dis 2005 64 396 402 15271773 10.1136/ard.2003.019711 PMC1755408

[B3] Wolfe F Anderson J Harkness D Bennett RM Caro XJ Goldenberg DL Russell IJ Yunus MB A prospective, longitudinal, multicenter study of service utilization and costs in fibromyalgia Arthritis Rheum 1997 40 1560 1570 10.1002/art.1780400904 9324009

[B4] Wolfe F Ross K Anderson J Russell IJ Hebert L The prevalence and characteristics of fibromyalgia in the general population Arthritis Rheum 1995 38 19 28 10.1002/art.1780380104 7818567

[B5] White KP Speechley M Harth M Ostbye T The London Fibromyalgia Epidemiology Study: the prevalence of fibromyalgia syndrome in London, Ontario J Rheumatol 1999 26 1570 1576 10405947

[B6] Haq SA Darmawan J Islam MN Uddin MZ Das BB Rahman F Chowdhury MA Alam MN Mahmud TA Chowdhury MR Tahir M Prevalence of rheumatic diseases and associated outcomes in rural and urban communities in Bangladesh: a COPCORD study J Rheumatol 2005 32 348 353 15693098

[B7] Campbell SM Clark S Tindall EA Forehand ME Bennett RM Clinical characteristics of fibrositis. I. A "blinded," controlled study of symptoms and tender points Arthritis Rheum 1983 26 817 824 10.1002/art.1780260701 6347207

[B8] Fries JF Spitz P Kraines RG Holman HR Measurement of patient outcome in arthritis Arthritis Rheum 1980 23 137 145 10.1002/art.1780230202 7362664

[B9] Meenan RF Mason JH Anderson JJ Guccione AA Kazis LE AIMS2. The content and properties of a revised and expanded arthritis impact measurement scales health status questionnaire Arthritis Rheum 1992 35 1 10 10.1002/art.1780350102 1731806

[B10] Burckhardt CS Clark SR Bennett RM The fibromyalgia impact questionnaire: development and validation J Rheumatol 1991 18 728 733 1865419

[B11] Bennett R The Fibromyalgia Impact Questionnaire (FIQ): a review of its development, current version, operating characteristics and uses Clin Exp Rheumatol 2005 23 S154 162 16273800

[B12] Bae SC Lee JH Cross-cultural adaptation and validation of the Korean fibromyalgia impact questionnaire in women patients with fibromyalgia for clinical research Qual Life Res 2004 13 857 861 15129895 10.1023/B:QURE.0000021688.92614.60

[B13] Buskila D Neumann L Assessing functional disability and health status of women with fibromyalgia: validation of a Hebrew version of the Fibromyalgia Impact Questionnaire J Rheumatol 1996 23 903 906 8724306

[B14] Hedin PJ Hamne M Burckhardt CS Engstrom-Laurent A The Fibromyalgia Impact Questionnaire, a Swedish translation of a new tool for evaluation of the fibromyalgia patient Scand J Rheumatol 1995 24 69 75 10.3109/03009749509099287 7747146

[B15] Offenbaecher M Waltz M Schoeps P Validation of a German version of the Fibromyalgia Impact Questionnaire (FIQ-G) J Rheumatol 2000 27 1984 1988 10955342

[B16] Perrot S Dumont D Guillemin F Pouchot J Coste J Quality of life in women with fibromyalgia syndrome: validation of the QIF, the French version of the fibromyalgia impact questionnaire J Rheumatol 2003 30 1054 1059 12734906

[B17] Rivera J Gonzalez T The Fibromyalgia Impact Questionnaire: a validated Spanish version to assess the health status in women with fibromyalgia Clin Exp Rheumatol 2004 22 554 560 15485007

[B18] Sarmer S Ergin S Yavuzer G The validity and reliability of the Turkish version of the Fibromyalgia Impact Questionnaire Rheumatol Int 2000 20 9 12 10.1007/s002960000077 11149662

[B19] Sarzi-Puttini P Atzeni F Fiorini T Panni B Randisi G Turiel M Carrabba M Validation of an Italian version of the Fibromyalgia Impact Questionnaire (FIQ-I) Clin Exp Rheumatol 2003 21 459 464 12942697

[B20] Zijlstra TR Taal E van de Laar MA Rasker JJ Validation of a Dutch translation of the fibromyalgia impact questionnaire Rheumatology (Oxford) 2007 46 131 134 10.1093/rheumatology/kel171 16757485

[B21] Kim YA Lee SS Park K Validation of a Korean version of the fibromyalgia impact questionnaire J Korean Med Sci 2002 17 220 224 11961307 10.3346/jkms.2002.17.2.220 PMC3054848

[B22] Dunkl PR Taylor AG McConnell GG Alfano AP Conaway MR Responsiveness of fibromyalgia clinical trial outcome measures J Rheumatol 2000 27 2683 2691 11093454

[B23] Bengali language Bengali language http://en.wikipedia.org/wiki/Bengali_language

[B24] Beaton DE Bombardier C Guillemin F Ferraz MB Guidelines for the process of cross-cultural adaptation of self-report measures Spine 2000 25 3186 3191 10.1097/00007632-200012150-00014 11124735

[B25] Wolfe F Smythe HA Yunus MB Bennett RM Bombardier C Goldenberg DL Tugwell P Campbell SM Abeles M Clark P The American college of rheumatology 1990 criteria for the classification of fibromyalgia. report of the multicenter criteria committee Arthritis Rheum 1990 33 160 172 10.1002/art.1780330203 2306288

[B26] Basak T Cross-cultural adaptation and validation of a Bengali health assessment questionnaire for use in rheumatoid arthritis patients. Thesis 2005 Dinajpur: Medical College, Department of Medicine 10.1111/1756-185X.12032 23992261

[B27] Feroz AH Islam MN ten Klooster PM Hasan M Rasker JJ Haq SA The Bengali SF-36 was acceptable, reliable, and valid in patients with rheumatoid arthritis J Clin Epidemiol 2012 In press 10.1016/j.jclinepi.2012.05.004 23017640

[B28] Lohr KN Assessing health status and quality-of-life instruments: Attributes and review criteria Qual Life Res 2002 11 193 10.1023/A:1015291021312 12074258

[B29] Ware JE The status of health assessment 1994 Annu Rev Public Health 1995 16 327 354 10.1146/annurev.pu.16.050195.001551 7639876

[B30] Guillemin F Bombardier C Beaton D Cross-cultural adaptation of health-related quality of life measures: literature review and proposed guidelines J Clin Epidemiol 1993 46 1417 1432 10.1016/0895-4356(93)90142-N 8263569

[B31] Wagner AK Gandek B Aaronson NK Acquadro C Alonso J Apolone G Bullinger M Bjorner J Fukuhara S Kaasa S Cross-cultural comparisons of the content of SF-36 translations across 10 countries: results from the IQOLA Project. International Quality of Life Assessment J Clin Epidemiol 1998 51 925 932 10.1016/S0895-4356(98)00083-3 9817109

[B32] Bennett RM Friend R Jones KD Ward R Han BK Ross RL The revised fibromyalgia impact questionnaire (FIQR): validation and psychometric properties Arthritis Res Ther 2009 11 R120 10.1186/ar2783 19664287 PMC2745803

[B33] Mease PJ Clauw DJ Christensen R Crofford LJ Gendreau RM Martin SA Simon LS Strand V Williams DA Arnold LM Toward development of a fibromyalgia responder index and disease activity score: OMERACT module update J Rheumatol 2011 38 1487 1495 10.3899/jrheum.110277 21724721 PMC3426365

[B34] Wolfe F Clauw DJ Fitzcharles MA Goldenberg DL Katz RS Mease P Russell AS Russell IJ Winfield JB Yunus MB The American College of Rheumatology preliminary diagnostic criteria for fibromyalgia and measurement of symptom severity Arthritis Care Res (Hoboken) 2010 62 600 610 10.1002/acr.20140 20461783

[B35] Jacobs JW Rasker JJ van der Heide A Boersma JW de Blecourt AC Griep EN van Rijswijk MH Bijlsma JW Lack of correlation between the mean tender point score and self-reported pain in fibromyalgia Arthritis Care Res 1996 9 105 111 10.1002/1529-0131(199604)9:2<105::AID-ANR1790090206>3.0.CO;2-# 8970268

[B36] Callahan LF Pincus T A clue from a self-report questionnaire to distinguish rheumatoid arthritis from noninflammatory diffuse musculoskeletal pain. The P-VAS:D-ADL ratio Arthritis Rheum 1990 33 1317 1322 10.1002/art.1780330903 2403397

