# Supplementary material for: Cross-cultural adaptation and validation of a Bengali version of the modified fibromyalgia impact questionnaire
Source: BMC Musculoskelet Disord. 2012 Aug 27;13:157. doi: 10.1186/1471-2474-13-157 (PMC3493366; doi:10.1186/1471-2474-13-157)
Supplement: Additional file 1 — Final version of the B-FIQ (Bengali and English items). [file 1471-2474-13-157-S1.doc]

**APPENDIX: Final version of the B-FIQ (Bengali and English items)**

bvg t ......................................................................................................... ZvwiL t ......../......./.........

Name: …………………………………………………………………..Date: ……./……/……

|  | wb‡`©kbv t 1 †_‡K 11 bs cÖ‡kœi Rb¨, `qv K‡i ‡mB msL¨vwU‡Z †Mvj `vM w`b hv Avcbvi MZ mßv‡ni mvwe©K ¯^v¯’¨ Ae¯’v fvjfv‡e eY©bv K‡i| hw` †Kvb cÖkœ Avcbvi Kg©Kv‡Ûi mv‡_ m¤ú„³ bv nq, Z‡e ev` w`b| | |
| --- | --- | --- |
|  | Directions: For questions 1 through 3, please circle the number that best describes how you did overall for the past week. If you don't normally do something that is asked, cross the question out. | |
|  |  |  |
| **1** | Avcwb wK †c‡i‡Qb t | Were you able to: |
|  | me mgq ‡ekxifvM mgq gv‡S gv‡S KLbI bv | Always Most times Occasionally Never |
|  |  |  |
|  |  |  |
|  | 1| †KbvKvUv (KvPv evRvi, Kvco †Pvci I cÖmvabx mn) Ki‡Z? | 1. Do shopping, including kitchen market, clothes and cosmetics? |
|  | 2| Kvco †Pvco ay‡Z? | 2. Wash clothes? |
|  | 3| Lvevi ˆZix (KzUvevQvmn ivbœv) Ki‡Z? | 3. Prepare food, including dressing and cooking? |
|  | 4| nvuwo cvwZj nvZ w`‡q ay‡Z? | 4. Wash dishes by hand? |
|  | 5| †g‡S Svo– w`‡q cwi¯‹vi Ki‡Z? | 5. Sweep floor with a broom? |
|  | 6| weQvbv (Pv`i, evwjk, Kvu_v, gkvixmn) †MvQv‡Z? | 6. Prepare beds, including bed cover, quilt, pillow and mosquito net? |
|  | 7| GK wK‡jvwgUv‡ii AwaK nuvU‡Z? | 7. Walk more than 1 km? |
|  | 8| eÜz-evÜe ev AvZœx‡qi evmvq †eov‡Z †h‡Z? | 8. Visit friends, relatives and neighbors? |
|  | 9| DVvb cwi¯‹vi (Svo– †`Iqv mn) Ki‡Z? | 9. Clean (including sweeping) the yard? |
|  | 10| ewU w`‡q ZiKvix KzU‡Z? | 10. Dress vegetable with the help of a ‘'boti’? |
|  | 11| wmuwo †e‡q DV‡Z? | 11. Climb stairs? |
|  |  |  |
| **2** | MZ mßv‡ni mvZ w`‡bi g‡a¨ KZ w`b Avcwb fvj †eva K‡i‡Qb?  ( 0 1 2 3 4 5 6 7 ) | |
|  | Of the 7 days in the past week, how many days did you feel good?  ( 0 1 2 3 4 5 6 7 ) | |
|  |  | |
| **3** | MZ mßv‡ni KZw`b Avcbvi ˆ`bw›`b KvR (PvKzix) Ges M„n¯’vjxi KvR dvB‡eªvg‡qjwRqvi Rb¨ Ki‡Z cv‡ib wb?  ( 0 1 2 3 4 5 6 7 ) | |
|  | How many days last week did you miss work, including housework, because of fibromyalgia?  ( 0 1 2 3 4 5 6 7 ) | |

|  | wb‡`k©bv t evKx cÖ‡kœi Rb¨ bx‡Pi ‡¯‹‡ji Dci †duvUv w`b hv Avcbvi MZ mßv‡ni mvgwMÖK Ae¯’v me‡P‡q fvjfv‡e wb‡`©k K‡i| |
| --- | --- |
|  | Directions: For the remaining items, mark the point on the line that best indicates how you felt overall for the past week. |
|  |  |
| **4** | Avcwb hLb KvR K‡i‡Qb, dvB‡eªvgv‡qjwRqvi e¨_v ev Ab¨vb¨ DcmM© Avcbvi M„n¯’vwji KvRmn mKj Kv‡R wK iKg weNœ NwU‡q‡Q?    Kv‡R †Kvb Amyweav nqwb Kv‡R LyeB ‡ekx Kó n‡q‡Q |
|  | When you worked, how much did pain or other symptoms of your fibromyalgia interfere with your ability to do your work, including housework? (No problem with work – Great difficulty with work) |
|  |  |
| **5** | Avcbvi e¨_v KZ Zxeª wQj?    †Kvb e¨_v wQj bv LyeB ‡ekx Zxeª e¨_v |
|  | How bad has your pain been? (No pain – Very severe pain) |
|  |  |
| **6** | Avcbx KZUzKy K¬všÍ wQ‡jb?    †Kvb K¬vwšÍ wQj bv LyeB ‡ekx K¬všÍ |
|  | How tired have you been? (No tiredness – Very tired) |
|  |  |
| **7** | mKv‡j Nyg †_‡K DVvi ci Avcwb †Kgb †eva K‡i‡Qb?    fv‡jv m‡ZR †eva K‡iwQ LyeB †ekx K¬všÍ-†j‡M‡Q |
|  | How have you felt when you get up in the morning? (Awoke well rested – Awoke very tired) |
|  |  |
| **8** | Avcbvi AvoóZv KZUzKz Lvivc wQj?    ‡Kvb AvoóZv wQjbv LyeB †ekx Avoó |
|  | How bad has your stiffness been? (No stiffness – Very stiff) |
|  |  |
| **9** | Avcwb KZUzKz DwØMœ wQ‡jb?    †Kvb D‡ØM wPj bv LyeB †ekx DwØMœ |
|  | How nervous or anxious have you felt? (Not anxious – Very anxious) |
|  |  |
| **10** | Avcwb KZUzKz welbœ †eva K‡i‡Qb?    welbœ wPjvg bv LyeB †ekx welbœ |
|  | How depressed or blue have you felt? (Not depressed – Very depressed) |
